# High-Density Lipoprotein Cholesterol Is a Favorable Prognostic Factor and Negatively Correlated with C-Reactive Protein Level in Non-Small Cell Lung Carcinoma

**DOI:** 10.1371/journal.pone.0091080

**Published:** 2014-03-13

**Authors:** Pei-Dong Chi, Wen Liu, Hao Chen, Jing-Ping Zhang, Yuehao Lin, Xin Zheng, Wanli Liu, Shuqin Dai

**Affiliations:** 1 State Key Laboratory of Oncology in Southern China, Sun Yat-sen University Cancer Center, Guangzhou, China; 2 Department of Clinical Laboratory Medicine, Sun Yat-sen University Cancer Center, Guangzhou, China; University of Bari & Consorzio Mario Negri Sud, Italy

## Abstract

**Background:**

Although the alterations of lipid profile in lung cancer have been documented, the prognostic value of serum HDL-C level and its correlation with inflammation in NSCLC remain unknown.

**Subjects and Methods:**

Levels of preoperative serum lipid concentrations (including HDL-C, LDL-C, TC, and TG) and the inflammatory biomarker C-reactive protein level (CRP) were retrospectively analyzed in 228 patients with NSCLC and in 300 healthy controls. The serum lipid levels in these two populations were compared. Univariate and multivariate cox hazards analyses were performed to investigate the prognostic value of serum lipid levels in NSCLC. The correlation between CRP and lipid profile were also analyzed.

**Results:**

Compared with those in normal controls, the serum HDL-C, LDL-C, and TC levels were statistically decreased and the TG levels were significantly increased in 228 NSCLC patients. The patients with decreased levels of HDL-C had significantly lower 5-year survival rates than those with normal HDL-C, not only in the whole NSCLC cohort but also in the subgroups stratified according to the disease T, N classifications, and metastasis, whereas the other lipid components were not independent prognostic factors for NSCLC. Of the lipid components, a lower HDL-C level was observed more often in patients with a high CRP level than in those with a normal CRP level. Spearman’s rank correlation analysis revealed that the HDL-C level presented a negative correlation with the CRP level (r = −0.360, *p*<0.001).

**Conclusions:**

A decreased level of preoperative HDL-C was found to be associated with poor survival in patients with NSCLC. Serum HDL-C level may be a clinical prognosis factor for NSCLC patients. In addition, a negative correlation was present between the levels of HDL-C and CRP, the well-known inflammation biomarker.

## Introduction

Non-small cell lung carcinoma (NSCLC), which accounts for 85% of the primary bronchogenic carcinomas, has the highest mortality rate of malignant tumors in the world [1]. Most patients who have been clinically diagnosed are already in a middle or advanced stage, and the 5-year survival rate is very low [2]. Although many serum biomarkers have been reported to predict the survival of NSCLC, the sensitivity and specificity are not sufficient and reliable [3]. Therefore, there is a growing need to identify an accurate biomarker to predict the outcome of NSCLC.

The role of lipid metabolism in cancer development has not been fully explored [4], [5], [6], [7], [8], [9]. Some researchers found that cholesterol synthesis is enhanced in cancer cells compared with normal cells, cancer cells need excess cholesterol and intermediates of the cholesterol biosynthesis pathway to maintain a high level of proliferation [10]. HDL lipoprotein may work as a supplier of cholesterol to cancer cells by removing excess cholesterol from peripheral tissues [10]. Beyond the known functions of cholesterol in the cell plasma membrane, it enters in the composition of lipid rafts, which are key elements in the signaling pathways of normal and cancer cells [11]. Modulation of cholesterol depletion of lipid rafts in vitro inhibited NSCLC cell migration through delocalization of the focal adhesion complex [12]. In large cohort studies, higher HDL-C concentration has been proved to be associated with a decreased risk of cancer overall, and in particular of lung cancer [13] while the prognostic role of HDL-C, LDL-C, TC and TG is not completely defined. HDL-C was found to be a risk and prognostic factor of prostate cancer [14], its prognostic value remains unclear in NSCLC.

Lipid metabolism changes not only from cancer but also during inflammation [15]. Chronic inflammation has been recognized as a key factor that contributes to the development and progression of a variety of malignancies [16]. Cytokines and C-reactive protein (CRP) are elevated during inflammation. Previous studies that investigated the changes in lipids during inflammation are predominantly focused on the relationship between the cytokines and the lipids [17], [18]. Those studies observed an increase of TG [19] and a decrease of HDL-C [20] during inflammation. Lim et al reported that chronic inflammation would reduce serum HDL-C concentrations [21], but the alterations of LDL-C and TC levels are controversial [22], [23]. By now, a few studies have focused on the relationship between the lipid profile and CRP, which is released by the liver in response to systemic infection or tissue damage.

In this study, we retrospectively investigated the prognostic values of the pre-therapy levels of the serum lipid profile, including HDL-C, LDL-C, TC, and TG in NSCLC. In addition, we analyzed the correlation of CRP with the lipid profile and smoking status in 228 patients with NSCLC, thereby evaluating the association of the lipids, inflammation, and NSCLC.

## Subjects and Methods

### Subjects

A total of 228 NSCLC patients with stage I through IV who were undergoing resection of primary cancer at the Sun Yat-sen University Cancer Center, Guangzhou, China, from April 2005 to December 2011 were enrolled in this study. All patients met the following eligible criteria: (1) all patients required a pathological confirmed diagnosis of NSCLC with no previous or coexisting cancer; (2) none had received therapy before serum collection; (3) patients with concomitant diseases that were associated with increasing serum lipid levels (i.e., diabetes, hyperlipidemia, or metabolic syndrome) were excluded; and (4) patients taking hormone replacement therapy or any drugs known to affect lipid metabolism were excluded. Overall, there were 170 men and 58 women with a mean age of 58 years (ranging from 33 to 91 years) enrolled in this study. The treatment of the patients was adherent to the national guideline (NCCN guideline Chinese version). Platinum-based neoadjuvant chemotherapy was performed for some patients (n = 67, stage III–IV) who were not suitable for surgery, until they reach the qualification to receive the surgery. The platinum-based chemotherapeutic regimen was mostly gemcitabine plus platinum, docetaxel plus platinum and paclitaxel plus platinum. 161 patients underwent primary lung tumor resection without receiving any preoperative treatment. The patients (n = 207, stage IB–IV) were offered consisted of at least 4 cycles of adjuvant platinum-based chemotherapeutic regimen after surgery, and only 21 cases (stage IA-IB) had not been offered the postoperative chemotherapy.

The EGFR mutation detection has been performed as the routine examination for the NSCLC patients since December of 2008 in our hospital. Totally, EGFR mutation detection was performed in 156 patients of the whole cohort. Only 54 patients were positive for EGFR mutation. The anti-EGFR drug (gefitinib and erlotinib) was used in 46 cases.

Detailed clinical and pathological information, including demographic data, smoking status, pathological tumor, node, metastasis stage and overall survival data were available for all patients. Tobacco index was calculated by the formula below: cigarettes per day multiplied by years of smoking; the cutoff value of tobacco index was 300 [24]. The nutritional status of the subjects was assessed by calculating the body mass index (BMI) (body weight in kg/height in meters^2^). The cutoff value of BMI was 25 according to the American Association of Clinical Endocrinologists (AACC) guideline [25].

According to the American Joint Committee on Cancer (AJCC) 7th Edition [26], the histologic type was classified into two groups: an SCC (squamous cell carcinoma) group (n = 82) and a non-SCC group (including adenocarcinoma, large cell carcinoma, and so on) (n = 146). Histologically, moderately and well-differentiated types were classified as differentiated carcinoma, and the low differentiated type was defined as undifferentiated carcinoma. The TNM stage of each carcinoma sample was determined according to the criteria of the WHO and the UICC TNM staging system (7^th^ edition) [27]. Overall patient survival, defined as the time from surgery to death or last follow up, whichever came first, was used as a measure of prognosis.

### Laboratory Measurements

Prior to use of these patients’ sera, written informed consents were obtained from each of the participants and the experiment was approved by the Institute Research Ethics Committee of Cancer Center of Sun Yat-Sen University, Guangzhou, China. Serum levels of HDL-C, LDL-C, TC, TG, and CRP were examined in early morning samples obtained before therapy and immediately measured using a Hitachi 7600-020 automatic biochemical analyzer. The TC was measured by the CHOD-PAP method on whole serum; HDL-C and LDL-C were detected by the selective elimination method (direct method) and selective protection method, respectively. Serum TG was measured by the GPO-PAP method. The test kits were all provided by Wako Pure Chemical Industries, Japan. The serum level of CRP was detected by the latex immunological transmission turbidimetry,and the kits were provided by SEKISUI Medical Co., LTD, Japan. A group of 300 normal subjects whose age and sex were matched during the same period was conducted as a control. We used an EGFR kit (GP Medical Technologies Ltd, Beijing, China) to detect a deletion mutation in exon 19 (delE746-A750 and delT747-P753>S) and point mutation in exon 21 (L858R and L861Q) with real time polymerase chain reaction. Positive results were defined as cycle threshold (Ct) ≤34 on the growth curve. The samples with positive results (34< Ct ≤38) were subjected to repeated RT-PCR for result validation.

There were no clearly defined cut points for lipids and CRP in relation to cancer outcomes; thus, we chose to use the cardiovascular clinical cut points to define the lipids and CRP levels because those have been extensively used in epidemiological research and would provide a well-defined criterion. According to the National Cholesterol and Education Program (NCEP) Adult Treatment Panel III criteria (NCEP-ATPIII) [28], a serum concentration of TC at 200 mg/dL or more, HDL-C less than 40 mg/dL in men or 50 mg/dL in women, LDL-C at 130 mg/dL or more, and TG at 150 mg/dL or greater were defined as hypercholesterolemia, categorical low HDL-C, high LDL-C, and hypertriglyceridemia. The eligible patients were divided into two groups: the low HDL-C group and the normal HDL-C group, according to the above cutoff value. We defined the cutoff value of serum CRP as 3.0 mg/L, which could be taken to define a state in a person without detectable causes other than inflammation [29], so we categorized the patients into two groups according to this cutoff value: the high CRP group (≥3.0 mg/L) and the normal CRP group (<3.0 mg/L). Moreover, we divided the whole cohort into four groups according to the cutoff values of serum HDL-C and CRP mentioned above. The criteria were listed below: low HDL-C and high CRP, group 1; normal HDL-C and high CRP, group 2; low HDL-C and normal CRP, group 3; and normal HDL-C and normal CRP, group 4.

### Statistical Analysis

All statistical analyses were performed using SPSS 17.0 statistical software package (SPSS Inc., Chicago, IL). The unpaired Student’s *t*-test was used to evaluate the difference in lipid levels between NSCLC patients and healthy controls. The unpaired Student’s *t*-tests and one-way ANOVA were used to analyze the association between HDL-C level and the observed clinical characteristics of patients. Pearson’s chi squared test was used to analyze the relationship between categorical HDL-C groups with clinicopathological features. The correlation of CRP levels with lipid concentrations and the smoking profile was analyzed by the Mann-Whitney U Test and χ2 test. The data with a normal distribution were expressed as the mean ± standard deviation, and the median (range) was used to express the data with a nonnormal distribution. The association between HDL-C and CRP was analyzed by Spearman’s rank correlation. The survival curves were plotted by the Kaplan-Meier method and compared using the log rank test. The significance of the variables for survival was analyzed using the Cox proportional hazards model (univariate and multivariate analysis). All the variables that were shown to be significant based on the univariate analysis were then examined using the multivariate Cox proportional hazard model to identify the independent variables that were correlated with the NSCLC mortality rate. All tests were two-tailed, and a *P*-value of <0.05 was considered to be significant.

## Results

### Pre-therapy Serum Levels of Lipids in NSCLC and Healthy Controls

**Figure 1:**
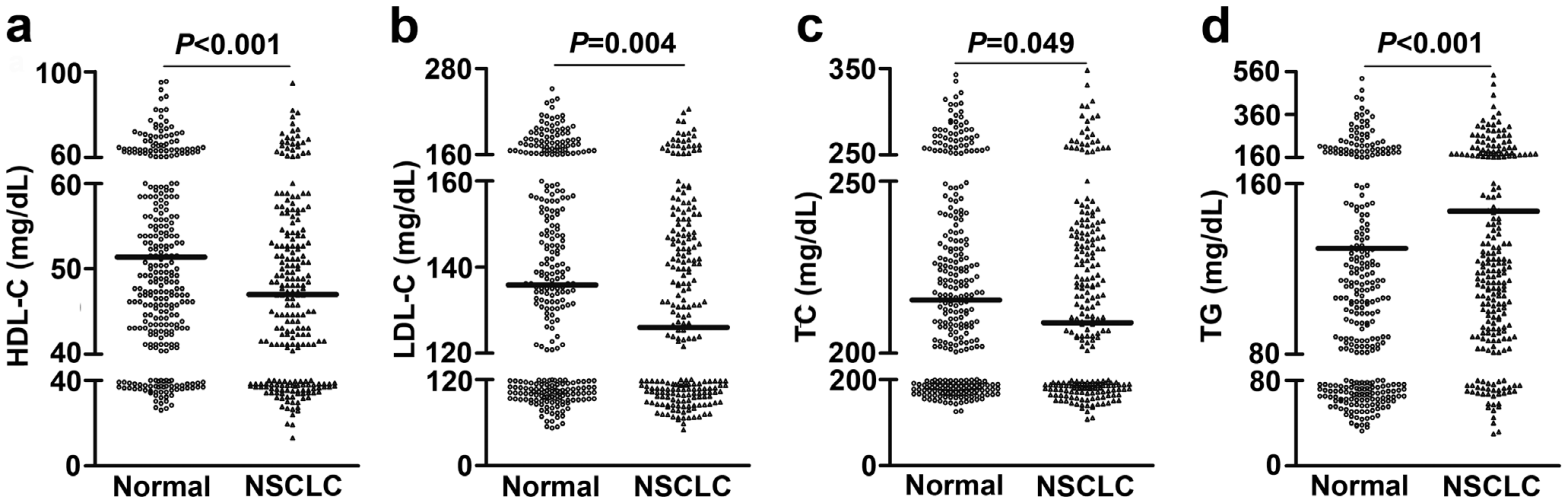
Pre-therapy serum levels of lipids in patients with NSCLC and in healthy controls. Pre-therapy serum levels of HDL-C (a), LDL-C (b), TC (c), and TG (d) in NSCLC and healthy controls. Each *dot* represents the lipid level from one patient or one control, and the line in the graph indicates the median value.

To investigate whether lipid abnormalities occur in NSCLC, the levels of lipids were compared between the healthy controls (n = 300) and NSCLC patients (n = 228) using the unpaired Student’s t-test (Figure 1). The pre-therapy serum levels of HDL-C (47.0±11.95 mg/dL), LDL-C (125.99±32.44 mg/dL), and TC (208.90±39.36 mg/dL) in NSCLC patients were significantly lower than those (HDL-C: 51.40±12.46 mg/dL; LDL-C: 135.82±37.20 mg/dL; TC: 215.44±39.40 mg/dL) in the age- and sex-matched normal controls. However, the level of TG (147.01±98.34 mg/dL) in NSCLC patients was significantly higher than that in healthy controls (129.60±105.34 mg/dL).

### Prognostic Significance of the Serum Lipid Profile in NSCLC

To evaluate the prognostic value of the lipids in NSCLC, the parameters, including demographic data, clinicopathological features, smoking status, preoperative chemotherapy, postoperative chemotherapy, anti-EGFR drug treatment, EGFR mutation status and lipid profile were evaluated by the univariate and multivariate Cox regression model (Table 1). The univariate analysis revealed that N classification, metastasis, TNM stage, tobacco history, tobacco index, CRP levels, and HDL-C levels had a significant association with NSCLC survival. To determine whether these seven factors could be used as an independent prognostic factor for survival, a multivariate analysis was carried out, based on TNM stage, tobacco index, CRP concentration, HDL-C level, age, and gender. The reason that we exclude the factors of N classification, metastasis, and tobacco history in the multivariate analysis is to eliminate the influence of statistical colinearity. Multivariate analysis showed that TNM stage (*p* = 0.004), tobacco index (*p* = 0.002), and HDL-C level (*p*<0.001) were independent prognostic indicators of the NSCLC survival. Thus, our findings indicated that the serum HDL-C level before therapy may serve as a novel independent prognostic factor for NSCLC.

**Table 1:** Univariate and multivariate cox hazards analysis for overall survival in 228 patients with NSCLC.

Variables	Univariate analysis			Multivariate analysis		
	HR	95%CI	*P*-value*	HR	95%CI	*P*-value*
Gender						
Male *vs.* Female	0.62	0.36–1.10	0.100	1.59	0.78–3.23	0.204
Age(years)						
<60 *vs.* ≥60	0.83	0.54–1.29	0.413	0.91	0.57–1.44	0.684
BMI**						
<25 *vs.* ≥25	0.72	0.33–1.56	0.400			
Histology typing						
SCC *vs.* non-SCC	1.27	0.91–1.77	0.169			
Histological differentiation						
Differentiated *vs.* Undifferentiated†	0.75	0.55–1.03	0.074			
pTclassification						
T3-4 *vs.* T1-2	1.47	0.92–2.33	0.105			
pNclassification						
Yes *vs.* No	1.76	1.04–2.97	0.036			
pMetastasis						
Yes *vs.* No	1.59	1.02–2.48	0.039			
pTNM stage††						
III–IV *vs.* I–II	2.25	1.22–4.15	0.010	2.46	1.33–4.55	0.004
Tobacco history						
Yes *vs.* No	2.26	1.43–3.58	0.001			
Tobacco index#						
≥300 *vs.* <300	2.13	1.37–3.32	0.001	2.37	1.37–4.09	0.002
Preoperative chemotherapy						
Yes *vs.* No	1.25	0.97–1.86	0.104			
Postoperative chemotherapy						
Yes *vs.* No	0.69	0.42–1.14	0.150			
anti-EGFR drug treatment						
Yes *vs.* No	0.81	0.49–1.35	0.421			
EGFR mutation status||						
Yes *vs.* No	0.718	0.41–1.26	0.245			
CRP (mg/L)						
≥3.0 *vs.* <3.0	2.22	1.18–4.21	0.014	1.77	0.91–3.44	0.093
HDL-C (mg/dL)§						
<40 *vs.* ≥40	2.64	1.70–4.12	<0.001	2.70	1.69–4.33	<0.001
LDL-C (mg/dL)						
<130 *vs.* ≥130	0.91	0.59–1.41	0.669			
TC (mg/dL)						
<200 *vs.* ≥200	0.78	0.50–1.22	0.275			
TG (mg/dL)						
<150 *vs.* ≥150	0.99	0.63–1.56	0.967			

HR, Hazard ratio; 95% CI, 95% confidence interval; SCC, squamous cell carcinoma; EGFR, Epidermal growth factor receptor; CRP, C-reactive protein; HDL-C, high-density lipoprotein cholesterol; LDL-C, low-density lipoprotein cholesterol; TC, total cholesterol; TG, triglyceride.

*Cox hazard regression model.

**BMI, body mass index, weight divided by height squared, kg/m^2^.

† Moderately and well-differentiated histologic types were classified as differentiated carcinoma, low.

differentiated histologic type was defined as undifferentiated carcinoma.

†† TNM denoted tumor-node-metastasis.

#Tobacco index indicated cigarettes per day multiplied years of smoking.

|| The EGFR mutation status was unknown in 72 patients of the whole cohort.

§ The cut-off value of HDL-C was 40 mg/dL in men or 50 mg/dL in women.

**Figure 2:**
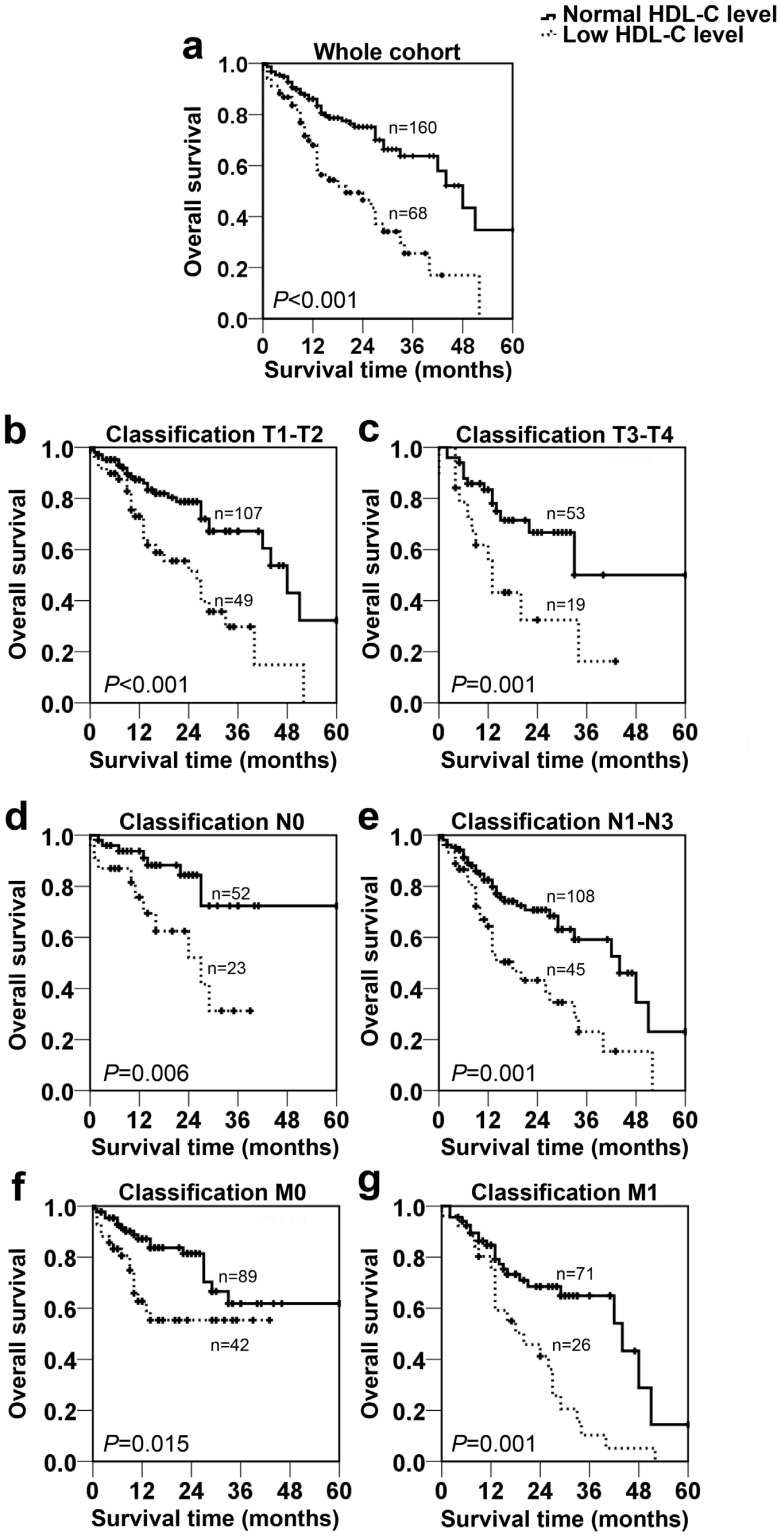
Prognostic significance of serum HDL-C in NSCLC. The patients were categorized into a low HDL-C group and a normal HDL-C group according to the cut-off value (40 mg/dl in men or 50 mg/dl in women). The five-year overall survival rate was calculated by the Kaplan-Meier method and analyzed by the log-rank test. HDL-C was a favorable prognostic factor in the whole NSCLC cohort (a), T1–T2 subgroup (b), T3–T4 subgroup (c), N0 subgroup (d), N1–N3 subgroup (e), M0 subgroup (f), and M1 subgroup (g).

**Figure 3:**
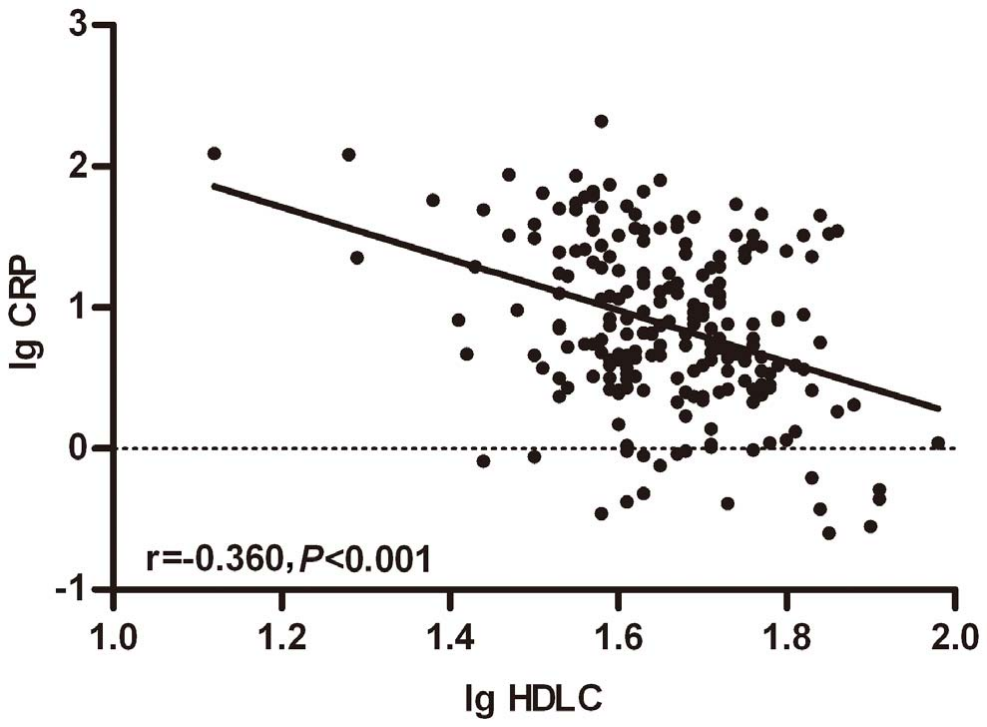
Correlation of serum HDL-C and CRP in 228 patients with NSCLC. The Pre-therapy serum HDL-C was negatively correlated with CRP (r = −0.360, p<0.001).

To further explore the prognostic significance of HDL-C level in NSCLC, the Kaplan-Meier method was performed to plot the survival curves, and the groups were compared using the log rank test. In the whole NSCLC cohort, patients with a normal HDL-C level (n = 160) showed a significantly better 5-year overall survival than the low HDL-C group (n = 68). The cumulative 5-year survival rate in the normal HDL-C group was 74.4%, whereas it was only 44.1% in the low HDL-C group (*p*<0.001, Figure 2a). We also analyzed the prognostic effect of the HDL-C level in selective patient subgroups stratified according to the disease T, N classifications, and metastasis, respectively. NSCLC patients with a decreased HDL-C level had a significantly shorter overall survival compared with those patients with a normal HDL-C level in the T1–T2 subgroup (n = 156, *p*<0.001, Figure 2b), the T3–T4 subgroup (n = 72, *p* = 0.001, Figure 2c), N0 subgroup (n = 75, *p* = 0.006, Figure 2d), N1–N3 subgroup (n = 153, *p* = 0.001, Figure 2e), M0 subgroup (n = 131, *p* = 0.015, Figure 3f), and M1 subgroup (n = 97, *p* = 0.001, Figure 2g).

### Relationship between HDL-C Level and Clinical Characteristics

The associations between median serum HDL-C levels and clinicopathological variables in 228 NSCLC patients are presented in Table 2. HDL-C levels were not associated with age, BMI, EGFR mutation status, T classification, N classification, metastasis, or TNM stage. In the entire cohort, women presented a significantly higher level of HDL-C compared the men (*p*<0.001). Decreased median HDL-C levels were significant for the patients with hypertriglyceridemia (*p*<0.001), elevated CRP level (*p*<0.001) and for those with a tobacco history (*p*<0.001) and a higher tobacco index (*p* = 0.008). The patients with squamous cell carcinoma (SCC) presented a lower level of HDL-C than those with adenocarcinoma (*p* = 0.027). There was also an association between decreased HDL-C levels and poorly differentiated tumors (*p*<0.001).

**Table 2:** Relationship between the HDL-C concentration and the clinical characteristics in 228 patients with NSCLC.

Variables	Cases	HDL-C(mg/dL)	*P*-value*	Normal HDL-C†	Low HDL-C†	*P*-value**
	(n)	(Mean±SD)		≥40 mg/dL	<40 mg/dL	
Number of cases	228			160	68	
Gender						
Male	170	44.66±10.75	<0.001	108	62	<0.001
Female	58	53.87±12.71		52	6	
Age (≥60 years)						
No	122	46.26±12.30	0.317	83	39	0.448
Yes	106	47.85±11.54		77	29	
BMI (≥25) ††						
No	121	47.11±11.98	0.381	85	36	0.980
Yes	107	46.24±11.95		75	32	
Hypertriglyceridemia||						
(≥150 mg/dL)						
No	156	48.85±12.47	<0.001	115	39	0.032
Yes	72	43.0±9.67		45	29	
CRP (≥3.0 mg/L)						
No	55	52.96±14.02	<0.001	46	9	0.012
Yes	173	45.11±10.57		114	59	
Tobacco history						
No	109	50.04±12.61	<0.001	84	25	0.031
Yes	119	44.22±10.62		76	43	
Tobacco index (≥300)§						
No	124	48.88±12.76	0.008	91	33	0.047
Yes	104	44.76±10.54		69	35	
EGFR mutation status#						
No	102	48.21±11.23	0.177	77	25	0.350
Yes	54	46.10±14.58		37	17	
Histology typing						
SCC	82	43.41±10.07	0.027	49	33	0.034
non-SCC	146	48.58±12.58		111	35	
Histological differentiation						
Low	86	45.08±10.67	<0.001	54	32	0.001
Moderate	99	44.65±11.48		66	33	
Well	43	56.24±11.22		40	3	
pTclassification						
T1	48	48.04±14.38	0.593	33	15	0.712
T2	108	48.57±11.92		74	34	
T3	33	47.59±12.86		26	7	
T4	39	45.89±10.57		27	12	
pNclassification						
No	75	47.15±12.19	0.793	52	23	0.846
Yes	153	46.70±11.53		108	45	
pMetastasis						
No	131	48.44±12.46	0.117	89	42	0.391
Yes	97	45.93±11.49		71	26	
pTNM stage‡						
I	38	48.33±12.50	0.506	25	13	0.628
II	26	46.42±12.29		16	10	
III	67	46.21±11.08		49	18	
IV	97	44.94±11.64		70	27	

Mean ± SD stands for Mean ± standard deviation; HDL-C, high-density lipoprotein cholesterol; CRP, C-reactive protein; EGFR, Epidermal growth factor receptor; SCC, squamous cell carcinoma.

*P values were calculated by the unpaired Student’s *t*-tests or one-way ANOVA, *P*<0.05 considered as statistically significant.

**P values were calculated by the chi-square test (χ2 test), *P*<0.05 considered as statistically significant.

† Low serum HDL-C was defined as <40 mg/dL in men or <50 mg/dL in women, the data above those values were classified as the normal HDL-C.

†† BMI, body mass index, weight divided by height squared kg/m^2^.

||The level of serum TG at 150 mg/dL or greater was defined as hypertriglyceridemia.

§ Tobacco index indicated cigarettes per day multiplied years of smoking.

#The EGFR mutation status was unknown in 72 patients of the whole cohort.

‡ TNM denoted tumor-node-metastasis.

Furthermore, the whole cohort was divided into two groups: normal HDL-C group and low HDL-C group, according to the HDL-C level (40 mg/dL in men or 50 mg/dL in women). The relationships between the categorical HDL-C groups and clinicopathological factors were assessed. Age, BMI, EGFR mutation status, T classification, N classification, the presence of metastasis, and TNM stage were similar in the two groups. Nevertheless, women presented a significantly lower rate in the low HDL-C group compared with the normal HDL-C group (*p*<0.001). The low HDL-C group had a significantly higher rate of hypertriglyceridemia (*p* = 0.032) and elevated CRP level (*p* = 0.012) compared with the normal HDL-C group. Patients with a tobacco history (*p* = 0.031), higher tobacco index (*p* = 0.047), and poor histological differentiation (*p* = 0.001) were more frequently observed in the low HDL-C group than in the normal HDL-C group. In addition, squamous cell carcinoma was observed more often in the patients from the low HDL-C group (*p* = 0.034).

### Correlation of CRP with Lipid Levels and Smoking Profile

The correlations of CRP with lipid levels and smoking profile in 228 NSCLC patients are presented in Table 3. CRP level was not associated with LDL-C, TC levels and EGFR mutation status. However, an elevated median CRP level was significant for the patients with a lower HDL-C level (*p*<0.001), tobacco history (*p* = 0.001), and higher tobacco index (*p*<0.001). The patients with hypertriglyceridemia presented a significantly lower level of CRP than those with a normal TG level (*p* = 0.006).

**Table 3:** Correlation of CRP concentration with lipid levels and smoking profile in 228 NSCLC patients.

Variables	Cases	CRP(mg/L)	*P*-value*	Normal CRP†	High CRP†	*P*-value**
	(n)	Median (Range)		<3.0 mg/L	≥3.0 mg/L	
Number of cases	228			55	173	
HDL-C (<40 mg/dL) ††						
No	160	5.39 (0.00–79.98)	<0.001	46	114	0.012
Yes	68	14.60 (0.00–206.78)		9	59	
LDL-C (≥130 mg/dL)						
No	121	6.62 (0.00–206.78)	0.794	29	92	0.953
Yes	107	6.45 (0.00–87.24)		26	81	
TC (≥200 mg/dL)						
No	93	8.24 (0.00–122.91)	0.188	19	74	0.279
Yes	135	5.53 (0.00–206.78)		36	99	
TG (≥150 mg/dL)						
No	156	9.04 (0.00–122.91)	0.006	35	121	0.381
Yes	72	4.59 (0.00–206.78)		20	52	
Tobacco history						
No	109	4.53 (0.00–63.98)	0.001	37	72	0.001
Yes	119	8.77 (0.35–206.78)		18	101	
Tobacco index (≥300) ||						
No	124	4.55 (0.00–87.24)	<0.001	40	84	0.002
Yes	104	10.02 (0.35–206.78)		15	89	
EGFR mutation status§						
No	102	5.57 (0.41–206.78)	0.620	25	77	0.489
Yes	54	7.86 (0.00–120.01)		16	38	

CRP, C-reactive protein; HDL-C, high-density lipoprotein cholesterol; LDL-C, low-density lipoprotein cholesterol; TC, total cholesterol; TG, triglyceride; EGFR, Epidermal growth factor receptor.

*P values were calculated by the Mann-Whitney U Test, P-value <0.05 considered as statistically significant.

**P values were calculated by the chi-square test (χ2 test), P-value <0.05 considered as statistically significant.

† High serum CRP was defined as >3.0 mg/L, the data below the cut off value were classified as the normal CRP.

†† The cut-off value of HDL-C was 40 mg/dL in men or 50 mg/dL in women.

||Tobacco index indicated cigarettes per day multiplied years of smoking.

§ The EGFR mutation status was unknown in 72 patients of the whole cohort.

Of the lipid components, only the low HDL-C level was observed more often in the patients from the high CRP group (*p* = 0.012); LDL-C, TC, TG and EGFR mutation status were similar in the categorized CRP groups. Furthermore, the patients with a smoking history (*p* = 0.001) and a higher tobacco index (*p* = 0.002) were observed more frequently in the high CRP group than in the normal CRP group. To confirm the relationship between the HDL-C and CRP, we performed the Spearman’s rank correlation analysis. The results revealed that the HDL-C and CRP presented a negative correlation (r = −0.360, *p*<0.001) (Figure 3).

### Prognostic Significance of Combining HDL-C and CRP

**Figure 4:**
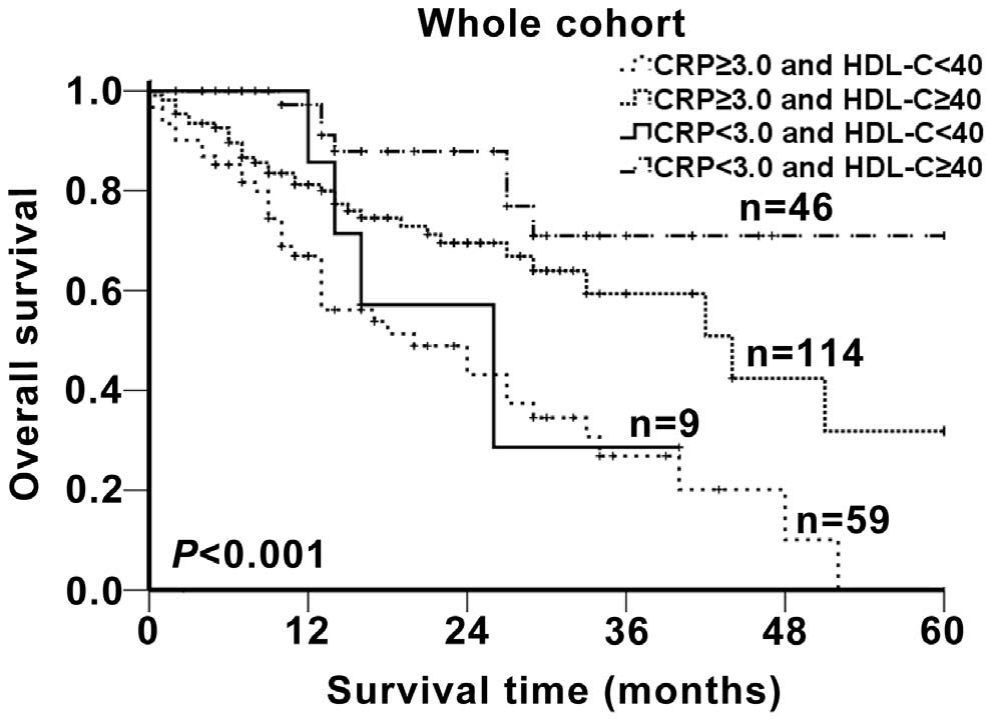
Prognosis significance of combining HDL-C and CRP. The patients were classified to four groups according to the cut-off values of HDLC (40 mg/dl in men or 50 mg/dl in women) and CRP (3.0 mg/L). Group 1: low HDL-C and high CRP; group 2: normal HDL-C and high CRP; group 3: low HDL-C and normal CRP; group 4: normal HDL-C and normal CRP. The five-years overall survival rates of the four groups were calculated by the Kaplan-Meier method and analyzed by the log-rank test.

The prognostic significance of combining HDL-C and CRP for the whole cohort was shown in Figure 4. The five-year overall survival rates of the four groups were significantly different (*p*<0.001). The patients with normal HDLC and normal CRP (group 4) had a significantly higher survival probability than the other three groups. Conversely, the survival rate of the patients with low HDL-C and high CRP (group 1) was the lowest among the four groups. The sequence of the survival rates of the four groups from high to low was group 4> group 2> group 3> group 1.

## Discussion

In this study, we found that levels of HDL-C, LDL-C, and TC were significantly lower, whereas TG was higher in patients with NSCLC compared with normal controls. Though the evidence of a certain association between serum lipid profile and the incidence of some cancers has been found in different ethnic groups and a large number of subjects [13], [21], [30], little is known about the prognosis effect of lipid components and cancer. It has been reported that hyperlipidemia is a negative prognosis factor for patients with gastric cancer and prostate cancer [14], [31]. However, few studies have been performed to examine the prognosis values of the lipid profile in NSCLC patients. Our study revealed that of the four lipid components (HDL-C, LDL-C, TC, and TG), only HDL-C was a prognostic indicator for NSCLC. In addition, we found that the patients with a low HDL-C level had a significantly shorter overall survival rate compared with those patients with a normal HDL-C level, not only in the whole cohort but also in the selective subgroups according to the disease T, N classifications, and metastasis.

The function of HDL-C in reverse cholesterol transport is very important to prevent the occurrence of atherosclerosis. However, the role of HDL-C in carcinogenesis is not well understood. The reason that the serum HDL-C level serves as a prognosis factor for NSCLC may be mainly attributed to its pleiotropic properties, including antioxidative function, inhibition of cytokine-induced expression of endothelial cell adhesion molecules, and suppression of the chemotactic activity of monocytes and lymphocytes [32], [33]. Recently, researchers used HDL-related apolipoproteins (ie, apolipoprotein A-I) and their mimetic peptides to observe the association between HDL and cancer cells. HDL mimetic peptide significantly reduced proliferation of colon cancer cells and the cell-mediated cancer burden in BALB/c mice [34]. The same mimetic peptide yielded similar results when tested against ovarian cancer [35]. These experimental studies can help explain the biological mechanisms for the association between HDL-C and NSCLC observed in our epidemiologic study. It’s well known that cholesterol, one of the components of HDL particle, plays a very important role in the cell plasma membrane. Beyond this, cholesterol is also known to accumulate in specific regions of the membrane, combined with sphingolipids creating small, compartmentalized, and highly stable micro-domains known as lipid-rafts which are referred to as the sites of signaling platforms that convey stimuli of the extracellular milieu to the intracellular systems in normal and cancer cells [10]. One study found that raft cholesterol depletion inhibited NSCLC cell migration through inhibition of phosphorylation of raft associated Src and dislocation of molecules comprising focal adhesion complexes from raft. This direct effect of cholesterol on NSCLC cell is evidence of a pathophysiologic relationship between HDL-C and NSCLC. All of these findings mentioned above revealed that HDL-C may serve as a protective agent in the development of cancer. Our results also suggest that the reduction of HDL-C levels that led to a reduction in the HDL-C functions may ultimately contribute to a milieu conductive to cancer development.

Epidermal Growth Factor Receptor (EGFR) is one of the receptor tyrosine kinase associated to lipid-raft and is involved in lung cancer [11]. Several small molecule tyrosine kinase inhibitors (TKIs) such as the anti-EGFR targeted drug gefitinib and erlotinib have been tested in clinical trials with some clinical success in lung cancer [36], [37]. These TKIs are aimed at the existing EGFR mutation in NSCLC cells, inhibit the tyrosine kinase activity of EGFR thus to suppress the growth of the cancer cells [38]. But the clinical efficacy of those anti-EGFR targeted drug in lung cancer is inconsistent. Current EGFR-targeted agents, including erlotinib and gefitinib, may result in dramatic responses, demonstrating efficacy in only a fraction of patients [39], [40]. New strategies to minimize the risk of resistance to EGFR inhibition have been employed in the development of next-generation EGFR tyrosine kinase inhibitors, such as PF00299804 and BIBW 2992 [40]. Some randomized study suggested that anti-EGFR targeted drug could only enhance the activity of chemotherapy but could not translate into longer overall survival [41]. Another clinical trial which recruited 1,217 East Asian with adenocarcinoma histology, never or light smoke randomized to receive the EGFR inhibitor gefitinib or carboplatin plus paclitaxel (CP) demonstrated that superior progression free survival and overall response rate occurred in the gefitinib group compared with CP but the overall survival was similar [42]. Our data displayed that the anti-EGFR drug (gefitinib and erlotinib) treatment was not associated with NSCLC overall survival by the univariate analysis (Table 1).

BMI is one of the indexes of nutrition. We noted that the HDL-C levels in the higher BMI group (≥25) and the normal BMI group (<25) were similar; BMI was not significantly different between the normal and low categorical HDL-C group (shown in table 2). These results indicated that nutritional status was not correlated with the HDL-C concentration. The lower level of HDL-C was not due to malnutrition in the NSCLC patients. The low level of HDL-C in malignancy might be explained by an increased demand for cholesterol from neoplastic cells. It is well known that the growth of tissues, including tumor tissues, utilizes the additional cholesterol to support membrane biosynthesis [9]. Because free cholesterol is preferentially diverted to storage as cholesterol esters in tumor cells, it is conceivable that the efflux of cholesterol is reduced during rapid tumor growth. This fact will lead to a reduction in the circulating levels of HDL-C. In particular, lower HDL-C levels accompanied higher CRP levels and a higher tobacco index in this study. The patients with a smoking history and a higher tobacco index presented a significantly decreased level of HDL-C and an increased level of CRP. Spearman’s rank correlation analysis revealed that the HDL-C and CRP presented a negative correlation. Epidemiologic studies suggest that chronic inflammation is involved in the development of cancers [16], including lung cancer [43]. The lung is a site for repeated and sometimes chronic inflammatory stimulus insults. One obvious stimulus is tobacco smoke, which contains numerous toxic chemicals and irritant particles that could lead to chronic inflammation; smoking is a well-documented and highly significant independent risk factor for lung cancer [44]. Furthermore, smoking has been shown to reduce serum HDL-C levels, possibly through induction of an inflammatory response and an associated increase in lipases that hydrolyze HDL-C and enhance clearance of HDL-C molecules from the bloodstream [45]. We found that HDL-C was negatively correlated with smoking status (including tobacco history and tobacco index) in NSCLC, providing further evidence to support this observation.

Although CRP was reported to be a prognostic factor in variable malignancy [46], no association between CRP and prognosis was observed in NSCLC. The finding may be attributed to the following reasons: (1) Pre-therapy serum CRP level may not be a valuable prognostic factor for NSCLC; (2) smoking and HDL-C are such strong prognostic factors for NSCLC that the prognostic effect of a relatively weaker risk factor, such as CRP, is not significant. The evidence supporting this conclusion was that CRP could predict prognosis immediately when HDL-C (Table S1) or tobacco index (Table S2) were excluded in the multivariate Cox hazard analysis.

Biomarkers that reflect ongoing inflammation conditions include the elevated proinflammatory cytokines, such as tumor necrosis factor-alpha (TNF) and interleukin-6 (IL-6), and elevated serum C-reactive protein (CRP) level [25]. Previous studies that investigated the changes in lipids during inflammation were predominantly focused on the relationship between cytokines and lipids [17], [18]. Those studies showed that the level of HDL-C will decrease, accompanied by elevated proinflammatory cytokines. In addition, chronic inflammation may also reduce the anti-inflammatory properties of HDL-C [15], [47]. In this study, we concentrated on the association between the CRP and lipid not only because of the convenient detection but also the innovation. In the cohort of this study, we observed that the patients who had a tobacco history and a higher tobacco index presented lower serum HDL-C levels and higher CRP levels compared with the non-smoker and lower tobacco index patients. It has been widely accepted that smoking could promote chronic pulmonary inflammation, leading to an increase in the serum CRP levels. In the present study, we found that increased CRP level was negatively correlated with reduced HDL-C level in NSCLC. The reduction of the HDL-C levels may lead to a reduction in the anti-inflammation functions of HDL-C, ultimately contributing to cancer development. Therefore, low serum HDL-C may serve as a clinical prognostic factor in NSCLC. The five-year overall survival rates of the patients with a low HDL-C level and a high CRP level were lowest compared with the other three groups (shown in Figure 4), whereas, the patients with normal HDLC and normal CRP had significantly higher survival rates among the four groups. These results have further affirmed the reversed relationship between HDL-C and CRP; moreover, they highlight the prognostic effect of the combining of HDL-C and CRP.

In summary, HDL-C concentration is an independent prognostic factor in NSCLC. This is confirmed also in some subgroups of patients according to the disease T, N classifications, and metastasis. There is currently a lack of prognostic markers in lung cancer. HDL-C presents a high reproducibility and it can easily be measured in all diagnostic laboratories. CRP alone as well as other inflammation markers are rarely able to predict prognosis. The use of two determinations, such as CRP and HLD-C, increases the prognostic significance of CRP alone. Perhaps in lung cancer it is preferable to use a system of two or more determinants to create a prognostic tool for clinicians, as with the use of albumin and beta2-microglobulin in multiple myeloma in the International Prognostic Score (ISS).

## Supporting Information

Table S1 Multivariate cox hazards analysis* for overall survival in 228 patients with NSCLC.
(DOCX)

Table S2 Multivariate cox hazards analysis* for overall survival in 228 patients with NSCLC.
(DOCX)
