# Supplementary material for: High-Density Lipoprotein Cholesterol Is a Favorable Prognostic Factor and Negatively Correlated with C-Reactive Protein Level in Non-Small Cell Lung Carcinoma
Source: PLoS One. 2014 Mar 13;9(3):e91080. doi: 10.1371/journal.pone.0091080 (PMC3953329; doi:10.1371/journal.pone.0091080)
Supplement: Table S1 — Multivariate cox hazards analysis* for overall survival in 228 patients with NSCLC. (DOCX) [file pone.0091080.s001.docx]

**Table S1．M**ultivariate cox hazards analysis* for overall survival in 228 patients with NSCLC

|  | HR | 95%CI | *P*-value** |
| --- | --- | --- | --- |
| Gender |  |  |  |
| Male *vs.* Female | 0.75 | 0.48-1.18 | 0.213 |
| Age(years) |  |  |  |
| <60 *vs.* ≥60 | 1.15 | 0.58-2.29 | 0.682 |
| pTNM stage† |  |  |  |
| Ⅰ-Ⅱ *vs.* Ⅲ-Ⅳ | 2.40 | 1.29-4.44 | 0.005 |
| Tobacco index†† |  |  |  |
| <300 *vs.* ≥300 | 2.13 | 1.24-3.67 | 0.006 |
| CRP (mg/L) |  |  |  |
| <3.0 *vs.* ≥3.0 | 2.06 | 1.06-4.0 | 0.032 |

HR, Hazard ratio; 95% CI, 95% confidence interval; CRP, C-reactive protein;

*HDL-C was excluded to eliminate the influence of statistical colinearity in this analysis.

**Cox hazard regression model.

†TNM denoted tumor-node-metastasis.

††Tobacco index was equal to cigarettes per day multiplies years of smoking.
