# Supplementary material for: High-Density Lipoprotein Cholesterol Is a Favorable Prognostic Factor and Negatively Correlated with C-Reactive Protein Level in Non-Small Cell Lung Carcinoma
Source: PLoS One. 2014 Mar 13;9(3):e91080. doi: 10.1371/journal.pone.0091080 (PMC3953329; doi:10.1371/journal.pone.0091080)
Supplement: Table S2 — Multivariate cox hazards analysis* for overall survival in 228 patients with NSCLC. (DOCX) [file pone.0091080.s002.docx]

**Table S2．M**ultivariate cox hazards analysis* for overall survival in 228 patients with NSCLC

|  | HR | 95%CI | *P*-value** |
| --- | --- | --- | --- |
| Gender |  |  |  |
| Male *vs.* Female | 0.93 | 0.52-1.69 | 0.821 |
| Age(years) |  |  |  |
| <60 *vs.* ≥60 | 0.99 | 0.63-1.56 | 0.961 |
| pTNM stage† |  |  |  |
| Ⅰ-Ⅱ *vs.* Ⅲ-Ⅳ | 2.47 | 1.33-4.58 | 0.004 |
| HDL-C (mg/dL) †† |  |  |  |
| < 40 *vs.* ≥ 40 | 2.51 | 1.57-4.02 | <0.001 |
| CRP (mg/L) |  |  |  |
| <3.0 *vs.* ≥3.0 | 1.94 | 1.0-3.74 | 0.042 |

HR, Hazard ratio; 95% CI, 95% confidence interval; HDL-C, high-density lipoprotein cholesterol; CRP, C-reactive protein.

*Tobacco index was excluded to eliminate the influence of statistical colinearity in this analysis.

**Cox hazard regression model.

†TNM denoted tumor-node-metastasis.

††The cut-off value of HDL-C was 40 mg/dL in men or 50 mg/dL in women.
